# Revisiting the Experimental Methods for Human Skin T-Cell Analysis

**DOI:** 10.1016/j.xjidi.2022.100125

**Published:** 2022-03-23

**Authors:** Takuya Sato, Youichi Ogawa, Aoha Ishikawa, Yuka Nagasaka, Manao Kinoshita, Ichiro Shiokawa, Shinji Shimada, Akira Momosawa, Tatsuyoshi Kawamura

**Affiliations:** 1 Department of Dermatology, Faculty of Medicine, University of Yamanashi, Yamanashi, Japan; 2 Department of Plastic Surgery, Faculty of Medicine, University of Yamanashi, Yamanashi, Japan

**Keywords:** T_RM_, resident memory T cells

## Abstract

Tissue-resident memory T cells exist in both the epidermis and the dermis in human skin. To analyze these cells, the skin needs to be incubated with dispase II to separate the two layers, that is, the epidermis and the dermis. The next step varies among researchers; the subsequent enzymatic digestion of the two layers is popular, whereas the spontaneous migration method can also be done. Scraping of these layers to yield skin T cells may reduce antigen modulation. This study aimed to determine each method’s limitations. Dispase II incubation itself cleaves T-cell antigens. Therefore, further enzymatic digestion with collagenases strongly cleaves antigens. The scraping method yields skin T cells that are affected by dispase II as it is. However, skin T-cell yield is low. The spontaneous migration method recovers and/or upregulates antigens with T-cell activation and loses ∼20% of T cells in the floating sheets. However, there was no prominent bias regarding CD103 expression between emigrants and the remaining T cells in the sheets. There were 10^4^ and 10^5^ CD3^+^ T cells per 1 cm^2^ of the epidermis and upper dermis, respectively. Collectively, each method has strengths and limitations to analyze both the epidermal and dermal T cells.

## Introduction

The abundant existence of tissue-resident memory T cells (T_RM_) not only in the dermis but also in the epidermis challenges researchers to reconsider the composition, phenotype, and function of human skin T cells (Clark et al., 2006b; Watanabe et al., 2015). However, owing to human skin samples’ limited availability, human skin T-cell extraction methods are controversial. Human skin in most studies is enzymatically digested with or without subsequent culture to restore enzymatically cleaved antigens. Trypsin or collagenase skin treatment has been known to cleave CD4 and other several antigens expressed on skin-resident antigen-presenting cells (Botting et al., 2017; Kashem and Kaplan, 2018; Lynch et al., 2003). Emigrants from skin explants are analyzed in other studies (Clark et al., 2006a; Cotton et al., 2021; McCully et al., 2018). In addition, the scraping method may reduce antigen modulation. In this study, we determined the number and phenotype of skin T cells in the epidermis and dermis, and we examined the limitations of these three methods: spontaneous migration method, enzymatic digestion method, and scraping method.

## Results

### Modulation of CD8 expression

**Figure 1:**
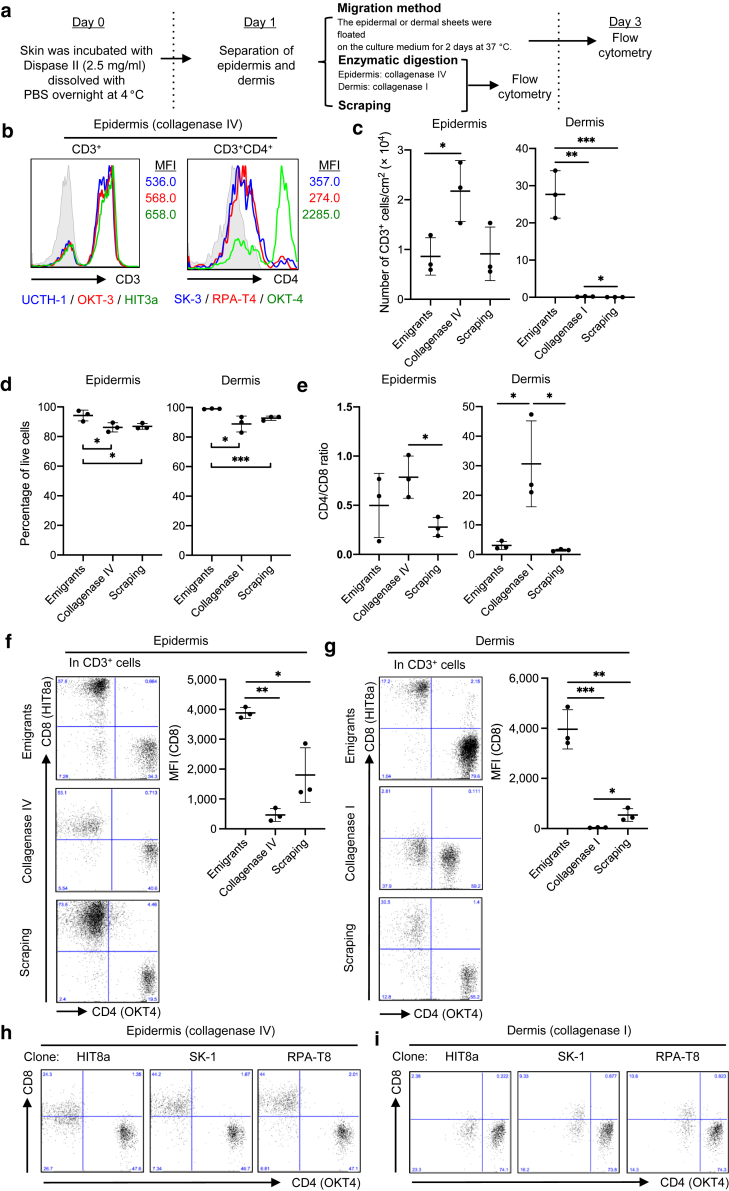
**Modulation of CD8 expression.** (**a**) A graphical figure showing the methodologies. (**b**) Representative histograms and MFI of total CD3^+^ and CD3^+^CD4^+^ cells with three different clones (n = 2). (**c**) Number of CD3^+^ T cells per cm^2^ of explants recovering by each method (n = 3). (**d**) Propidium iodide‒negative CD3^+^ T cells in each method (n = 3). (**e**) CD4-to-CD8 ratio of CD3^+^ T cells in each method (n = 3). (**f, g**) Representative data of CD4 and CD8 expression in (**f**) epidermal and (**g**) dermal CD3^+^ T cells (left). A summary of CD8 MFI (right; n = 3). (**h, i**) Representative data of CD4 and CD8 expression in (**h**) epidermal and (**i**) dermal CD3^+^ T cells with three different CD8 clones (n = 2). Comparisons between groups were evaluated using Student’s *t*-test (one tailed). ∗*P* < 0.05, ∗∗*P* < 0.01, and ∗∗∗*P* < 0.001. MFI, mean fluorescent intensity.

Institutional approval was obtained for all experiments that used human materials, and written informed consent was obtained from all subjects. The epidermal sheets were separated from the underlying upper dermis after dispase II incubation at 4 °C overnight. The epidermis and dermis were subjected to either spontaneous migration, enzymatic digestion, or scraping method. In the spontaneous migration method, the explants were floated on a medium without the addition of any exogenous cytokines and stimulations for 2 days at 37 °C, and then emigrants from the explants were analyzed. In the enzymatic digestion method, the epidermis and dermis were digested with collagenase type IV and collagenase type I, respectively, because dermal digestion requires enzymes with stronger trypsin activity than that of the epidermis. In the scraping method, explants were crammed between 100-μm nylon mesh sheets and gently scraped with forceps (Figure 1a).

On epidermal digestion with collagenase type IV, CD4 but not CD3 was completely cleaved, except for the epitope recognized by clone OKT-4, which was thereafter used for anti-CD4 antibodies, as previously reported (Botting et al., 2017; Lynch et al., 2003) (Figure 1b). First, CD3^+^ T-cell yield, CD3^+^ T-cell viability, and CD4-to-CD8 ratio in CD3^+^ T cells isolated by each method were examined. The enzymatic digestion method was superior in epidermal CD3^+^ T-cell yield to both the migration and scraping methods. However, the enzymatic digestion method and the scraping method were ineffective in yielding dermal CD3^+^ T cells (Figure 1c). The viability of the recovered epidermal and dermal CD3^+^ T cells was marginally but significantly retained in the migration method (Figure 1d). The enzymatic digestion method significantly increased the CD4-to-CD8 ratio of the recovered epidermal and dermal CD3^+^ T cells, indicative of CD8 antigen cleavage during the process (Figure 1e). As expected, enzymatic digestion of the epidermal (Figure 1f) and dermal (Figure 1g) sheets impaired CD8 expression, with varying degrees between CD8 clones (Figure 1h and i). On the contrary, assuming that the scraping method damages antigens less, the migration method significantly increased CD8 expression of epidermal (Figure 1f) and dermal (Figure 1g) CD3^+^ T cells, indicative of antigen recovery and/or upregulation during the 2-day culture on the medium. Collectively, the enzymatic digestion method cleaves CD8 antigen, whereas the migration method upregulates it in both the epidermal and dermal CD3^+^ T cells.

### Modulation of T_RM_ markers

**Figure 2:**
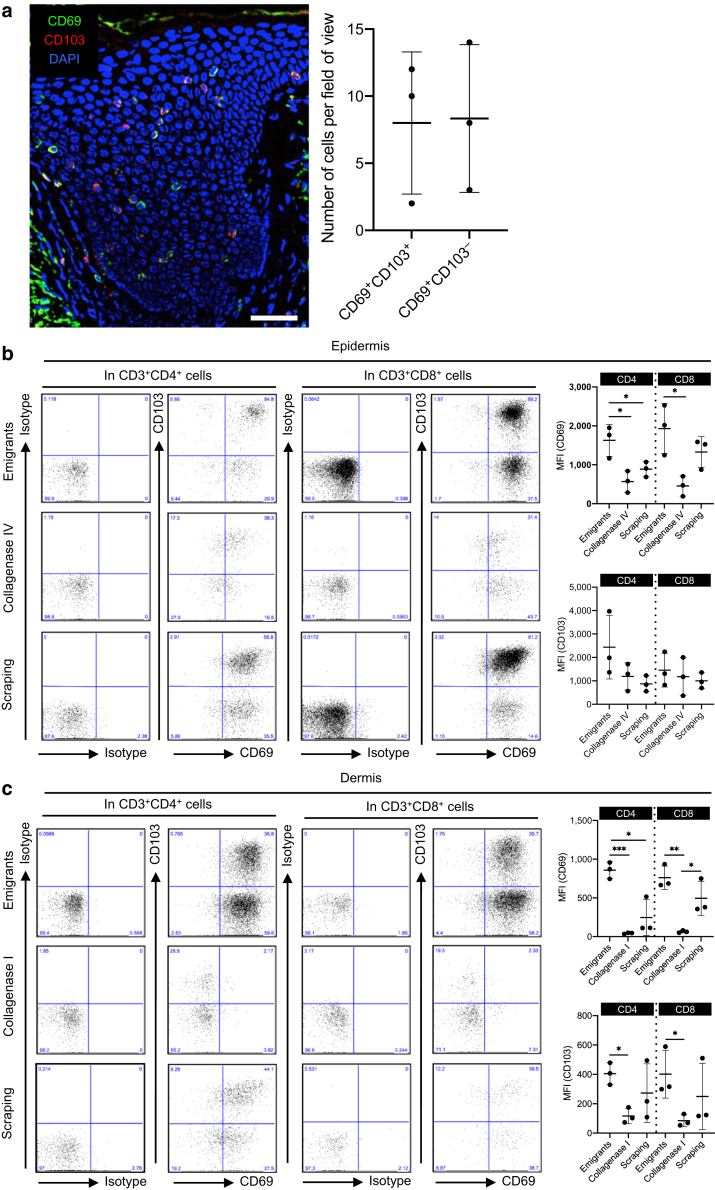
**Modulation of T_RM_ markers.** (**a**) A representative immunofluorescence image of CD69 (green), CD103 (red), and DAPI (blue) in healthy, noninflamed human skin. Bar = 30 μm (left). A summary of the number of each population (right; n = 3). (**b, c**) Representative data of CD69 and CD103 expression in (**b**) epidermal and (**c**) dermal CD3^+^CD4^+^ (left panels) or CD3^+^CD8^+^ T cells (middle panels). A summary of MFI of CD69 (upper right) and CD103 (lower right) (n = 3). Comparisons between groups were evaluated using Student’s *t*-test (one tailed). ∗*P* < 0.05, ∗∗*P* < 0.01, and ∗∗∗*P* < 0.001. MFI, mean fluorescent intensity; T_RM_, resident memory T cells.

The human epidermis from healthy volunteers contains small populations of CD69^+^ cells with or without CD103, with varying degrees between them (Figure 2a). Again, assuming that the scraping method damages antigens less, the enzymatic digestion method tended to cleave CD69, a T_RM_ marker, on both the CD4^+^ and CD8^+^ T cells of the epidermis (Figure 2b) and dermis (Figure 2c). As was the case for CD8, the migration method increased CD69 expression (Figure 2b and c). CD103, another T_RM_ marker, was less modulated on both the CD4^+^ and CD8^+^ T cells of the epidermis (Figure 2b) and dermis (Figure 2c). These data suggest that the enzymatic digestion method cleaves CD69 antigen, whereas the migration method upregulates it in both the epidermal and dermal CD4^+^ and CD8^+^ T cells.

### Migration method‒induced T-cell activation

**Figure 3:**
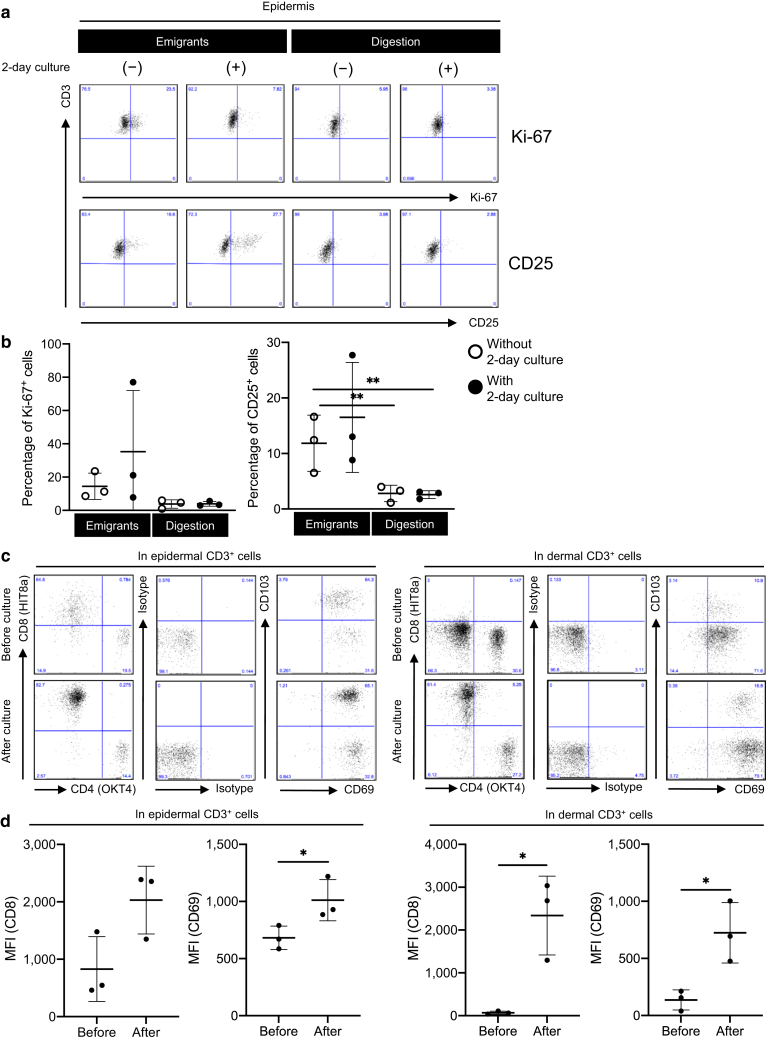
**Migration method‒induced T-cell activation.** (**a**) Representative expression of Ki-67 (upper) and CD25 (lower) in the live epidermal CD3^+^ cells with (second and fourth columns) or without (first and third columns) 2-day culture. (**b**) A summary of percentage of Ki-67^+^ (left) and CD25^+^ (right) cells in the live epidermal CD3^+^ cells (n = 3). Comparisons between groups were evaluated using two-way ANOVA test. ∗∗*P* < 0.01. (**c**) Representative data of CD4 and CD8 expression and CD69 and CD103 expression in epidermal (left panels) and dermal (right panels) CD3^+^ T cells before (upper panels) and after (lower panels) 2-day culture. (**d**) A summary of CD8 and CD69 MFI of epidermal (left) and dermal (right) CD3^+^ cells before (upper panels) and after (lower panels) 2-day culture (n = 3). Comparisons between groups were evaluated using Student’s *t*-test (one tailed). ∗*P* < 0.05. MFI, mean fluorescent intensity.

To confirm the notion that the migration method induces T-cell activation, epidermal CD3^+^ T cells were recovered by both the migration method and the enzymatic digestion method. Subsequently, Ki-67 and CD25 expression in these epidermal CD3^+^ T cells was determined. CD25 but not Ki-67 was significantly upregulated in the CD3^+^ T cells recovered by the migration method compared with those recovered by the enzymatic digestion method (Figure 3a, first and third columns, and Figure 3b, white circles). The subsequent 2-day culture of recovered CD3^+^ T cells in the culture medium upregulated the Ki-67 and CD25 expression in the CD3^+^ T cells recovered by the migration method but not in those recovered by the enzymatic digestion method (Figure 3a, second and fourth columns, and Figure 3b, black circles).

After a 2-day culture of dead cell‒excluded enzymatically digested epidermal and dermal single-cell suspension, the enzymatically cleaved CD8 and CD69 were restored (Figure 3c and d). Given that the 2-day culture of enzymatically digested epidermal single-cell suspension did not induce T-cell activation (Figure 3a and b), CD8 and CD69 expression recovery during a 2-day culture may not mediate T-cell activation. These data confirm that the migration method induces T-cell activation.

### The effect of collagenases

**Figure 4:**
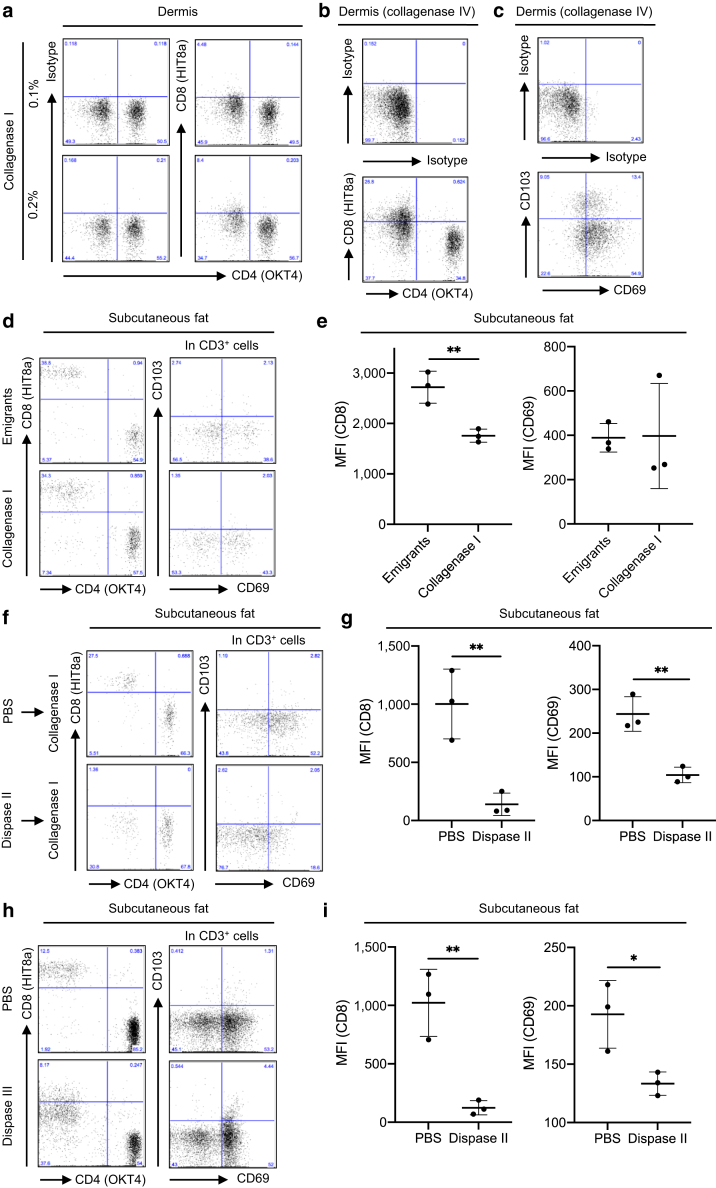
**The effect of collagenases.** (**a**) Representative data of CD4 and CD8 expression in dermal CD3^+^ T cells recovered with two different concentrations of collagenase type I (n = 2). (**b, c**) Representative data of (**b**) CD4 and CD8 expression and (**c**) CD69 and CD103 expression in dermal CD3^+^ T cells recovered with collagenase type IV (n = 2). (**d**) Representative data of CD4 and CD8 expression (left panels) and CD69 and CD103 expression (right panels) in subcutaneous fat CD3^+^ T cells recovered with either migration method or collagenase type I digestion. (**e**) A summary of MFI of CD8 (left) and CD69 (right) in subcutaneous fat CD3^+^ cells (n = 3). (**f**) Subcutaneous fat was incubated with PBS or dispase II overnight at 4 °C and then digested with collagenase type I. Representative data of CD4 and CD8 expression (left panels) and CD69 and CD103 expression (right panels) in subcutaneous fat CD3^+^ T cells. (**g**) A summary of MFI of CD8 (left) and CD69 (right) in subcutaneous fat CD3^+^ cells (n = 3). (**h**) Representative data of CD4 and CD8 expression (left panels) and CD69 and CD103 expression (right panels) in subcutaneous fat CD3^+^ T cells recovered with PBS or dispase II incubation overnight at 4 °C. (**i**) A summary of MFI of CD8 (left) and CD69 (right) in subcutaneous fat CD3^+^ cells (n = 3). Comparisons between groups were evaluated using Student’s *t*-test (one tailed). ∗∗*P* < 0.01. MFI, mean fluorescent intensity.

Collagenase type I strongly cleaved CD8 and CD69 of dermal T cells (Figure 1, Figure 2c), and even halving its concentration did not affect CD8 cleavage degree (Figure 4a). Dermal digestion with collagenase type IV instead of type I still impaired the CD8 (Figure 4b) and CD69 (Figure 4c) expressions. Dispase II incubation of the skin is crucial to separate the epidermis and dermis, but we can obtain subcutaneous fat directly from skin specimens without it. CD8 expression was impaired in the subcutaneous fat digested using collagenase type I compared with that in subcutaneous fat emigrants (Figure 4d and e). However, CD8 cleavage degree was apparently milder than that in the dermal enzymatic digestion in the combination of dispase II and collagenase type I (Figure 1g). Moreover, CD69 expression was comparable between subcutaneous fat emigrants and subcutaneous fat digested using collagenase type I (Figure 4d and e). To confirm the concept that the two-step enzyme usage such as dispase II incubation followed by collagenase-based digestion might cleave CD8 and CD69, subcutaneous fat was incubated with PBS or dispase II overnight at 4 °C and then digested with collagenase type I; combining dispase II and collagenase type I resulted in strong CD8 and CD69 cleavage (Figure 4f and g). Finally, subcutaneous fat was incubated with PBS or dispase II alone overnight at 4 °C, and then CD8 and CD69 expression was examined. Dispase II incubation alone cleaved CD8 and CD69 (Figure 4h and i) stronger than the one-step collagenase digestion (Figure 4d and e). Taken together, the effect of one-step collagenase digestion on antigen cleavage might be minimum. However, the two-step enzyme usage such as dispase II incubation followed by collagenase-based digestion might cleave many and unspecified antigens.

### The effect of dispase II

**Figure 5:**
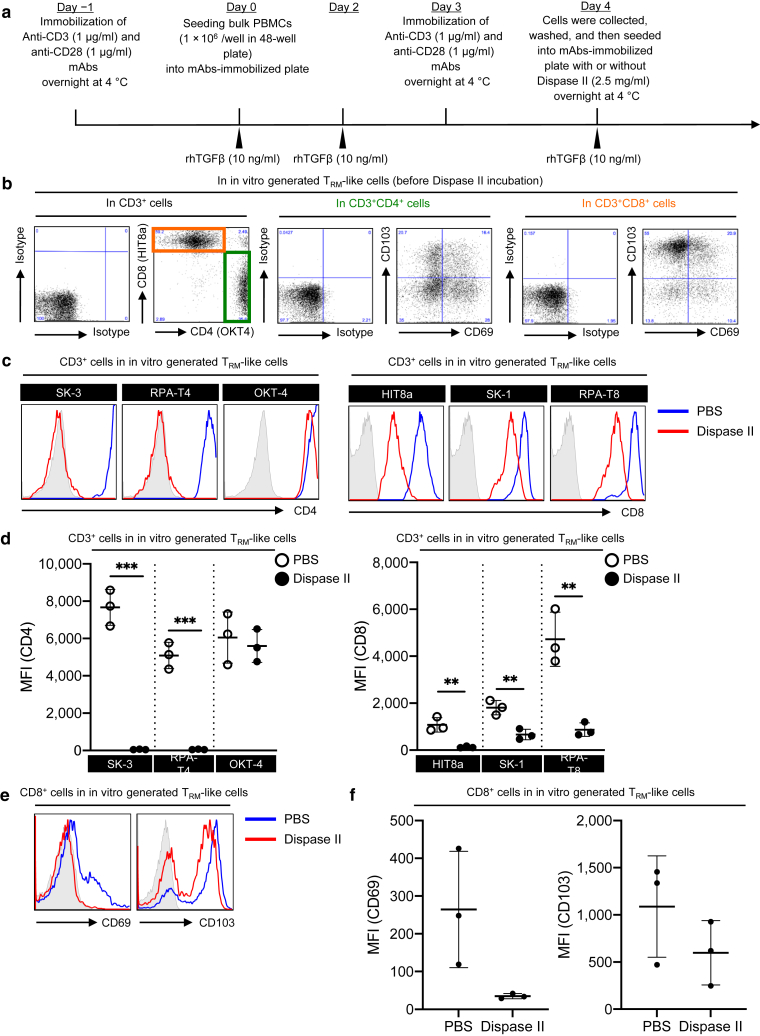
**The effect of dispase II.** (**a**) Time course of in vitro generation of T_RM_-like T cells from PBMCs. (**b**) Representative expression of indicated antigens in in vitro generated T_RM_-like T cells before dispase II incubation (n = 3). (**c, e**) Representative expression of indicated antigens in in vitro generated T_RM_-like T cells after dispase II incubation. (**d, f**) A summary of MFI of the indicated antigens in in vitro generated T_RM_-like T cells after dispase II incubation (n = 3). Comparisons between groups were evaluated using Student’s *t*-test (one tailed). ∗∗*P* < 0.01 and ∗∗∗*P* < 0.001. MFI, mean fluorescent intensity; rhTGFβ, recombinant human TGFβ; T_RM_, resident memory T cells.

As previously reported (Abuzakouk et al., 1996; Gedye et al., 2014), dispase II incubation cleaved CD8 and CD69 antigens. To examine its direct effects on CD8 and CD69 expression as well as CD4 and CD103 expression, CD69- and CD103-expressing T_RM_-like T cells were generated from PBMCs (Figure 5a and b), followed by dispase II incubation overnight at 4 °C. CD4 was completely cleaved, except for the epitope recognized by clone OKT-4 (Figure 5c and d, left), whereas CD8 was cleaved with varying degrees between CD8 clones (Figure 5c and d, right). Moreover, CD69 and CD103 expression on the CD8^+^ T cells also tended to be cleaved (Figure 5e and f). These data suggest that dispase II incubation alone could cleave T-cell markers (CD4 and CD8) and T_RM_ markers (CD69 and CD103).

### Comparison between emigrants and the remaining T cells in the explants

**Figure 6:**
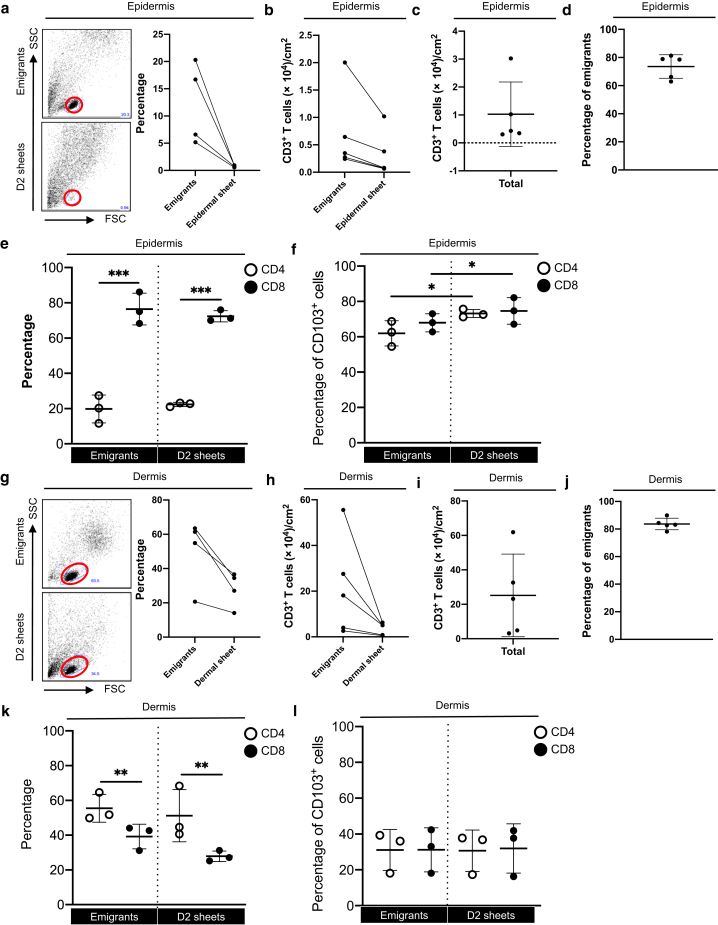
**Comparison between emigrants and the remaining T cells in the explants.** Representative data of FSC versus SSC gating for (**a**) epidermis and (**g**) dermis. Lymphocyte populations are indicated by red circles (left panels). A summary of the percentage of lymphocytes (right panels; n = 4). A summary of absolute CD3^+^ T-cell count per 1 cm^2^ of explants in (**b, c**) epidermis and (**h, i**) dermis (n = 5). A summary of the percentage of emigrants in the 1 cm^2^ of explants in (**d**) epidermis and (**j**) dermis (n = 5). Summaries of the percentage of CD4^+^ or CD8^+^ cells in CD3^+^ cells for (**e**) epidermis and (**k**) dermis) (n = 3). Summaries of the percentage of CD103^+^ cells in (**f**) epidermis and (**l**) dermis (n = 3). Comparisons between groups were evaluated using two-way ANOVA test. ∗*P* < 0.05, ∗∗*P* < 0.01, and ∗∗∗*P* < 0.001. D2, day 2; FSC, forward scatter; SSC, side scatter.

To validate the possibility that some biased T cells migrated from the epidermal and dermal sheets as emigrants in the migration method, a 2-day floating epidermal and dermal sheets were digested with collagenase types IV and I, respectively. The forward scatter/side scatter gating lymphocyte population was clearer in the emigrants than in the digested day 2 epidermal sheets because keratinocyte contamination was less in the emigrants (Figure 6a). The absolute CD3^+^ T-cell count per 1 cm^2^ of the epidermis was consistently higher in the emigrants than in the digested day 2 epidermal sheets (Figure 6b). The combined absolute CD3^+^ T-cell count of the emigrants and of the digested day 2 epidermal sheets, representing the total number of CD3^+^ T cells in 1 cm^2^ of the epidermis, was approximately 1 × 10^4^ (Figure 6c). Regardless of the number of CD3^+^ T cells residing in the epidermis, approximately 80% of CD3^+^ T cells migrated as emigrants (Figure 6d). CD8^+^ T cells were greater than CD4^+^ T cells in both the emigrants and the digested day 2 epidermal sheets (Figure 6e). CD103 expression in CD4^+^ and CD8^+^ T cells of both the emigrants and the digested day 2 epidermal sheets was approximately 70% (Figure 6f) and was marginally but significantly higher in the digested day 2 epidermal sheets than in the emigrants (Figure 6f).

Regarding the dermis, the forward scatter/side scatter gating lymphocyte population was comparably evident between emigrants and digested day 2 dermal sheets (Figure 6g). Similar to the epidermis, the absolute CD3^+^ T-cell count per 1 cm^2^ of the upper dermis was consistently higher in the emigrants than in the digested day 2 dermal sheets (Figure 6h). The combined absolute CD3^+^ T-cell count of the emigrants and the digested day 2 dermal sheets was approximately 2 × 10^5^ (Figure 6i), and regardless of the number of CD3^+^ T cells residing in the dermis, approximately 80% of CD3^+^ T cells migrated as emigrants (Figure 6j). Compared with the epidermis, CD4^+^ T cells were greater than CD8^+^ T cells in both the emigrants and the digested day 2 dermal sheets (Figure 6k). Moreover, CD103 expression in the CD4^+^ and CD8^+^ T cells of both the emigrants and the digested day 2 dermal sheets was comparable. Of note, because approximately 30% of dermal T cells expressed CD103, its CD103 expression was lower than that in the epidermal T cells (Figure 6l). These data suggest that the makeup of CD3^+^ T cells in the migration method is not skewed. In addition, migration efficacy might partially depend on CD103 in the epidermis but not in the dermis. Moreover, CD4^+^ and CD8^+^ T-cell composition in CD3^+^ T cells as well as the CD103 expression levels are quite different between the epidermis and dermis.

## Discussion

**Figure 7:**
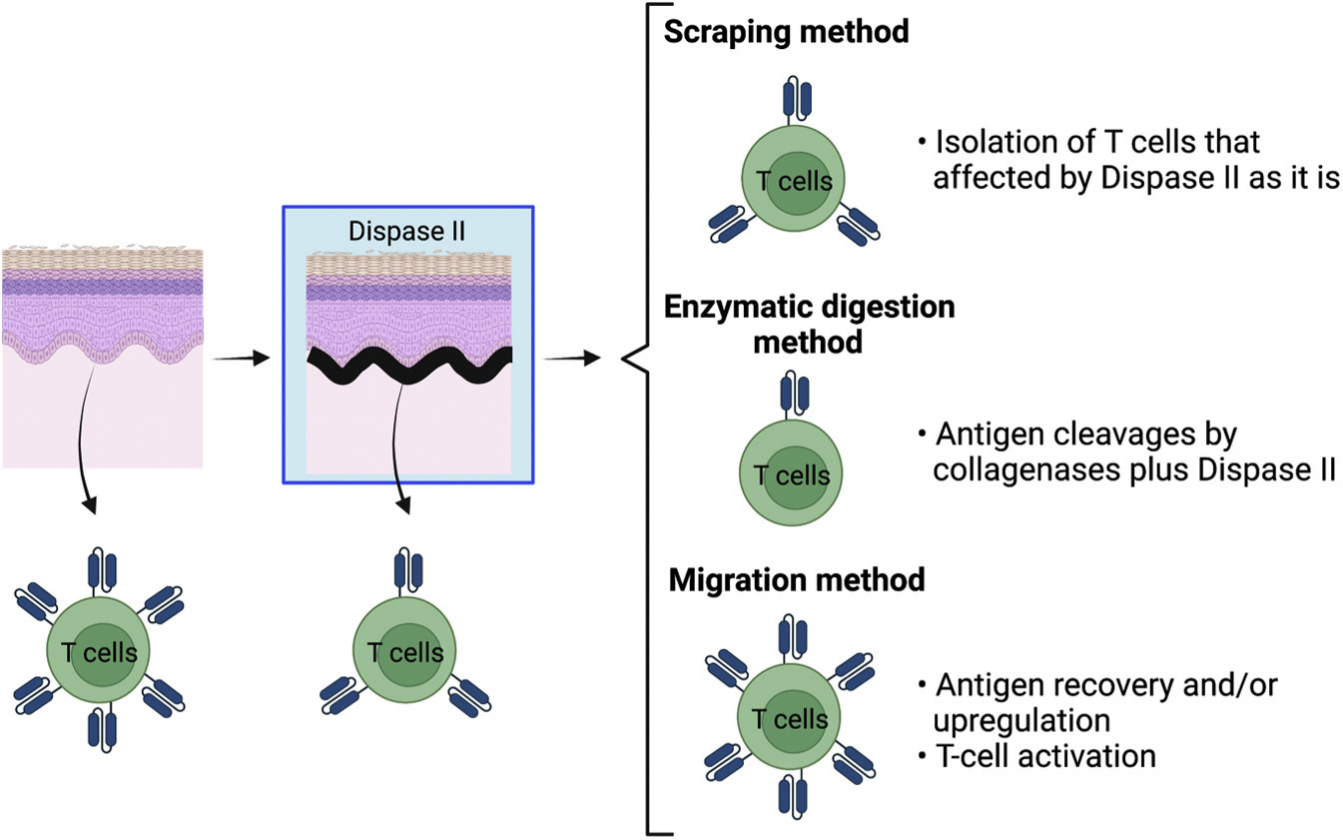
**Graphical summary of the characteristics of the three methods.** Dispase II incubation alone could cleave T-cell markers as well as T_RM_ markers. The scraping method is expected to yield the most natural skin T cells because these skin T cells are subject to be influenced by dispase II only. Digestion with an enzyme combination of dispase II and collagenases strongly cleaves CD4, CD8, and CD69 on skin T cells. The migration method could recover and/or upregulate antigens. However, these antigen expressions may be unnatural because this method induces T-cell activation. T_RM_, resident memory T cells.

There is no definitively superior method to analyze both the epidermal and dermal T cells whose antigen expressions are not modulated because dispase II incubation alone could cleave T-cell markers and T_RM_ markers. However, human skin T-cell analysis right after digestion with an enzyme combination of dispase II and collagenases should be avoided because it strongly cleaves CD4, CD8, and CD69 on skin T cells. Cleaved antigens are restored after a 2-day culture of digested single-cell suspensions without T-cell activation. However, in cases wherein a 2-day culture is needed to catch solid antigen expressions, the spontaneous migration and scraping methods take less time and resources. The scraping method is expected to yield the most natural skin T cells because these skin T cells are subject to be influenced by dispase II only. Epidermal (Figure 1, Figure 2b) and dermal (Figure 1, Figure 2c) T cells recovered by this method appeared to be less influenced by dispase II and sustain both the T-cell and T_RM_ markers than subcutaneous fat T cells (Figure 4h), possibly because epidermal and dermal T cells are protected by keratinocytes and fibroblasts, respectively. The only problem is the significantly lower skin T-cell yield, particularly in the dermis. Conversely, this method might be ideal in the case wherein only epidermal T cells are targeted. On the contrary, the migration method could recover and/or upregulate antigens. However, these antigen expressions may be unnatural because this method induces T-cell activation. Considering skin T-cell well-recovery efficacy from both the epidermis and dermis, this method might be useful to analyze skin T cells while keeping in mind the induced T-cell activation consistent with the recently published report (Kim et al., 2021) (Figure 7).

Using the migration method, we determine that there are 10^4^ and 10^5^ CD3^+^ T cells per 1 cm^2^ of the epidermis and upper dermis, respectively. In the epidermis, CD8^+^ T cells are greater than CD4^+^ T cells and vice versa in the dermis, and CD103 expression is higher in the epidermis than in the dermis. Given that TGFβ induces CD103 expression (Casey et al., 2012; El-Asady et al., 2005), the in situ levels of active-form TGFβ may be more abundant in the epidermis than in the dermis. The migration method loses ∼20% of T cells in the floating sheets. In the epidermis, CD103 expression was marginally higher in the digested day 2 epidermal sheets than in the emigrants, which is reasonable because CD103 binds to E-cadherin expressed on the epidermal keratinocytes and facilitates T_RM_ retention in situ (Hardenberg et al., 2018; Mackay et al., 2013; Moreau et al., 2021; Pauls et al., 2001). On the contrary, in the dermis, CD103 expression was comparable between emigrants and the remaining T cells in the sheets, which may indicate comparable retention ability between CD103^+^ and CD103^−^ T cells in the dermis.

## Materials and Methods

### Sources of tissues and ethical approval

The Institutional Review Board of the University Hospital (University of Yamanashi, Yamanashi, Japan) approved the acquisition of human tissues, and informed consent was obtained from all skin donors. Healthy, noninflamed human skin was obtained from routinely discarded tissue after plastic surgeries.

### Tissue processing

RPMI 1640 (Invitrogen Life Technologies, Carlsbad, CA) containing 10% fetal bovine serum (Biowest, Nuaillé, France) and Antibiotic-Antimycotic (1:100; Gibco, Dublin, Ireland) without additional exogenous cytokines was utilized as a culture medium. This study analyzed cells from the epidermis, dermis, and subcutaneous fat recovered through enzymatic digestion method, spontaneous migration method, or scraping method. On day 0, the skin was washed with sterile cold PBS (Gibco) immediately after surgery.

#### Subcutaneous fat T cells

To obtain emigrants, subcutaneous fat was removed and put in the culture medium for 2 days at 37 °C and then analyzed on day 2. For enzymatic digestion, subcutaneous fat was placed in PBS (Gibco) containing 2.5 mg/ml dispase II (Roche Diagnostics, Indianapolis, IN) overnight at 4 °C and/or 0.15% collagenase type Ⅰ (Worthington Biochemical, Lakewood, NJ) for 60 minutes at 37 °C.

#### Epidermal and dermal T cells

To obtain epidermal and dermal sheets, the subcutaneous fat and deep dermis were thoroughly removed using a pair of scissors. The parts of the skin composed of the epidermis and upper dermis were cut into approximately 20 × 10 mm square pieces and then incubated with dispase II (2.5 mg/ml; Roche Diagnostics) dissolved with PBS (Gibco) overnight at 4 °C. On day 1, to make a single-cell suspension from the epidermis and dermis, the epidermal sheets were incubated with collagenase type IV (200 U/ml; Worthington Biochemical) for 30 minutes at 37 °C, whereas the dermal sheets were incubated with PBS solution containing 0.2% collagenase type Ⅰ (Worthington Biochemical) and 0.05% DNase I (Sigma-Aldrich, St. Louis, MO) for 120 minutes at 37 °C. After incubation, the epidermal and dermal sheets were divided into small pieces using a pair of forceps. To generate single-cell suspensions, these pieces were aspirated with a 50-cc syringe up and down 5 times for epidermal suspension and 10 times for the dermal and subcutaneous fat suspension. This was then filtered thrice through a sterile mesh, with subsequent analysis on the same day (day 1). To obtain emigrants, the epidermal or dermal sheets were floated separately in the culture medium for 2 days at 37 °C. On day 3, these emigrants were analyzed.

### Flow cytometry

The following mAbs were used in the study: anti-CD3 (clones UCTH-1, OKT-3, and HIT3a), anti-CD4 (clones SK-3, RPA-T4, and OKT-4), anti-CD8a (clones HIT8a and RPA-T8), anti-CD8 (clone SK-1), anti-CD69 (clone FN50), anti-CD103 (clone Ber-ACT8), and anti-CD25 (clone M-A251). These mAbs were purchased from BioLegend (San Diego, CA). Cells were incubated with mAbs for 30 minutes at 4 °C and then washed twice in staining buffer and examined by FACS Caliber (BD Biosciences, Franklin Lakes, NJ). Dead cells were labeled with propidium iodide (Sigma-Aldrich). Data were analyzed using the FlowJo software (Tree Star, Ashland, OR). For Ki-67 staining, surface antigens were stained as described earlier, followed by cell fixation and permeabilization using eBioscience Foxp3/Transcription Factor Staining Buffer Set (Invitrogen, Carlsbad, CA) according to the manufacturer’s instructions. Cells were incubated with anti‒Ki-67 mAbs (clone 11F6; eBioscience, San Diego, CA) for 30 minutes at 4 °C and then washed twice in the buffer.

### Immunofluorescence

Human skin samples were fixed in 4% paraformaldehyde (Electron Microscopy Sciences, Hatfield, PA) for 24 hours at 4 °C, dehydrated in 30% sucrose (Sigma-Aldrich) for 24 hours at 4 °C, and then frozen in an embedding compound (Sakura Finetek, Tokyo, Japan) on dry ice. Next, 5-μm sections were rehydrated in 0.3% Triton X-100 (Pharmacia Biotech, Uppsala, Sweden) and blocked with 3% goat serum (Abcam, Cambridge, United Kingdom) for 1 hour. The sections were incubated with mouse anti-human CD69 mAbs (2.5 μg/ml; BD Biosciences) and rabbit anti-human CD103 mAbs (2.5 μg/ml; Abcam) overnight at 4 °C. After washing with 0.3% Triton X-100, the sections were incubated for 3 hours at room temperature with the following secondary antibodies: Alexa Fluor 488‒conjugated goat anti-mouse IgG and Alexa Fluor 647‒conjugated goat anti-rabbit IgG (1:500; Life Technologies, Carlsbad, CA). The sections were mounted with VECTASHIELD Mounting Medium supplemented with DAPI (Vector Laboratories, Burlingame, CA). Immunofluorescence images were obtained through fluorescence microscopy (BIOREVO BZ-9000; Keyence, Osaka, Japan). The cell number of CD69^+^CD103^+^ and CD69^+^CD103^−^ was counted in the 10 randomized areas (×400 magnification) of three sections each from different normal skin samples, and then the average cell number in each section was determined and summarized.

### CD3^+^ T-cell count

The sizes of the epidermal and dermal sheets were measured. The number of emigrants from the epidermal and dermal sheets was counted using a counting chamber, and then the percentage of CD3^+^ T cells among these was determined by flow cytometry. The day 2 epidermal and dermal sheets were digested with collagenase type IV and type I, respectively, after 2 days of floating. The number of total digested cells was counted using a counting chamber, and then the percentage of CD3^+^ T cells among these was determined by flow cytometry.

### Generation of T_RM_-like cells in vitro

Before PBMC culture (day ‒1), 1 μg/ml of anti-CD3 (clone UCHT1; BioLegend) and anti-CD28 (clone CD28.2; BioLegend) mAbs were immobilized to a 48-well culture plate overnight at 4 °C. On day 0, bulk PBMCs were isolated from peripheral blood of healthy volunteers using ficoll-paque plus (Cytiva, Uppsala, Sweden) and then seeded into mAbs-immobilized plate at a density of 1 × 10^6^ per well with the addition of 10 ng/ml human platelet‒derived TGFβ1 (R&D Systems, Minneapolis, MN). After 2-day culture at 37 °C, 10 ng/ml human platelet‒derived TGFβ1 (R&D Systems) was again added to the culture (day 2). On day 3, a culture plate‒immobilized 1 μg/ml of anti-CD3 and anti-CD28 mAbs was prepared. On day 4, cultures were collected, washed, and then reseeded into mAbs-immobilized plate at a density of 1 × 10^6^ per well, with an addition of 10 ng/ml human platelet‒derived TGFβ1 (R&D Systems) in the presence or absence of 2.5 mg/ml dispase II (Roche Diagnostics) overnight at 4 °C.

### Statistical analysis

Statistical analysis was conducted using Student’s *t*-test (one tailed) or two-way ANOVA test. Difference of ∗*P* < 0.05, ∗∗*P* < 0.01, and ∗∗∗*P* < 0.001 were considered statistically significant.

### Data availability statement

Datasets related to this article can be found at https://data.mendeley.com/datasets/n487348fbj/1, https://doi.org/10.17632/n487348fbj.1, hosted at INNOV-0201-0107.

## ORCIDs

Takuya Sato: http://orcid.org/0000-0001-7173-1677

Youichi Ogawa: http://orcid.org/0000-0003-2635-888X

Aoha Ishikawa: http://orcid.org/0000-0001-9083-2756

Yuka Nagasaka: http://orcid.org/0000-0002-1019-7885

Manao Kinoshita: http://orcid.org/0000-0002-0485-9803

Ichiro Shiokawa: http://orcid.org/0000-0001-7263-3622

Shinji Shimada: http://orcid.org/0000-0001-6387-3202

Akira Momosawa: http://orcid.org/0000-0003-1261-8441

Tatsuyoshi Kawamura: http://orcid.org/0000-0003-4008-2322

## Author Contributions

Conceptualization: YO, SS, TK; Investigation: TS, YO, AI, YN, MK, IS, AM; Writing - Original Draft Preparation: YO; Writing - Review and Editing: TS, YO, AI, YN, MK, IS, SS, AM, TK.

## Conflict of Interest

The authors state no conflict of interest.
